# Expression of Intermediate Filaments in the Developing Testis and Testicular Germ Cell Cancer

**DOI:** 10.3390/cancers14225479

**Published:** 2022-11-08

**Authors:** Maria E. Camacho-Moll, Leendert H. J. Looijenga, Roland Donat, Chitranjan J. Shukla, Anne Jørgensen, Rod T. Mitchell

**Affiliations:** 1 Department of Molecular Biology, Northeast Centre for Biomedical Research, Mexican Institute for Social Security, 2 de abril 501, Esq. San Luis Potosi, Col. Independencia, Monterrey C.P. 64720, Nuevo León, Mexico; 2 Princess Máxima Center for Pediatric Oncology, Heidelberglaan 25, 3584 CS Utrecht, The Netherlands; 3 Department of Urology, Western General Hospital, Crewe Road, Edinburgh EH4 2XU, Scotland, UK; 4 Department of Growth and Reproduction, University Hospital of Copenhagen, Rigshospitalet, Blegdamsvej 9, 2100 Copenhagen, Denmark; 5 MRC Centre for Reproductive Health, Queen’s Medical Research Institute, The University of Edinburgh, 47 Little France Crescent, Edinburgh EH16 4TJ, Scotland, UK

**Keywords:** Sertoli cells, human testis, TGCC, GCNIS, intermediate filaments, desmin, cytokeratin

## Abstract

**Simple Summary:**

Testicular germ cell cancer (TGCC) is the most common type of cancer among men between 15–44 years of age. This cancer originates in fetal life where normal germ cells, which ultimately become spermatozoa, fail to mature and instead remain in the testis in an immature state. These cells start the formation of tumours in early adulthood. Other cells which are in close contact with germ cells and are important for their survival and normal development and differentiation are the Sertoli cells. Sertoli cells are also immature in TGCC. We investigated the expression of two proteins; desmin and cytokeratin, which normally maintain tissue shape, in the Sertoli cells of normal testis and TGCC testis and we conclude that desmin is not useful to detect immature Sertoli cells whereas cytokeratin is expressed in Sertoli cells of tubules of TGCC patients and might be useful to diagnose patients with impaired testicular development and pre-invasive TGCC.

**Abstract:**

Cytokeratin and desmin expression have been associated with Sertoli cell maturity and the development of testicular germ cell cancer (TGCC). Thus, the present study aimed to characterize the expression of these intermediate filaments in normal testis development and TGCC. Cytokeratin and desmin were determined by immunohistochemistry and immunofluorescence in human fetal, and adult testis and tissue from patients with pre-invasive germ cell neoplasia in-situ (GCNIS) or invasive TGCC. Desmin was expressed in Sertoli cells of the human fetal testis, and the proportion of desmin expressing Sertoli cells was significantly reduced in the second trimester, compared with the first trimester (31.14% vs. 6.74%, *p =* 0.0016). Additionally, Desmin was expressed in the majority of Sertoli cells in the adult testis and TGCC samples. Cytokeratin was detected in Sertoli cells of human fetal testis but was not expressed in Sertoli cells of human adult testis. In patients with TGCC, cytokeratin was not expressed in Sertoli cells in tubules with active spermatogenesis but was detected in Sertoli cells in tubules containing GCNIS cells in patients with both pre-invasive and invasive TGCC. In conclusion, desmin was not associated with Sertoli cell maturation or progression to TGCC. However, cytokeratin appeared to be an indicator of impaired Sertoli cell maturation.

## 1. Introduction

Testicular germ cell cancer (TGCC) is the most common type of neoplasm in men between 15–44 years of age [1]. TGCC originates from fetal germ cells termed gonocytes which are arrested in differentiation at some point during development [2]. Normally the gonocytes differentiate to spermatogonia starting from the second trimester of fetal development but in TGCC, gonocytes arrest in this early stage and transform into pre-invasive germ cell neoplasia in-situ (GCNIS) cells, which remain dormant in the testis until around puberty when they form invasive TGCC. Testicular germ cell tumours in males are pathologically and clinically subdivided into seminomas and non-seminoma. Within the seminiferous tubules, besides germ cells, there are Sertoli cells, which normally support germ cell development. The cytoskeleton of Sertoli cells is composed of actin filaments, intermediate filaments, and microtubules, which have distinct patterns of distribution during the spermatogenic cycle [3,4]. The function of intermediate filaments in Sertoli cells is not entirely clear; however, the changes in their configuration during normal and abnormal spermatogenesis suggest a role in supporting tissue structure [4]. The intermediate filaments of the mature Sertoli cells are mainly of the vimentin type [4] and desmin and cytokeratin, which are also intermediate filaments, have been associated with immature Sertoli cells and impaired spermatogenesis [5,6,7,8,9,10,11,12]. In TGCC, desmin and cytokeratin expression have been reported in Sertoli cells [9,11]. The present study aimed to characterise desmin and cytokeratin expression in Sertoli cells of normal testis in fetal life, adulthood, and in the progression from pre-invasive GCNIS to invasive TGCC. 

## 2. Materials and Methods

### 2.1. Tissue Collection

*Human fetal testis tissue*: Following elective termination of pregnancy, women gave informed consent and human fetal testicular tissue of 9 to 20 weeks of gestation (n = 36) was obtained. Human fetal testis tissue was collected with ethical approval under the project registered with the REC reference: LREC08/1101/1 and 08/H0906/21+5. Ultrasound and foot length measurement were used to determine gestational age and qPCR was performed to determine the expression of sex-determining region gene Y (SRY) for sex determination as previously described [13]. Details about the proteins analysed in these samples can be seen in Table A1.

*Normal adult testicular tissue*: Normal testicular tissue with complete spermatogenesis (n = 6; age 47–52 years) was obtained from patients undergoing orchidectomy for a variety of clinical indications such as chronic pain, previous trauma, and post-vasectomy pain. For pathological assessment, tissue was fixed in formalin. Ethical approval was obtained for the use of archived human testicular tissue from the pathology department at the Western General Hospital in Edinburgh (REC Reference: 10/S1402/33) and from the biobank at the Department of Growth and Reproduction, University Hospital of Copenhagen, Denmark (H-1-2012-007). Details about the proteins analysed in these samples can be seen in Table A2.

TGCC samples: Tissue from children with pre-invasive TGCC (pre-GCNIS cells or GCNIS cells without evidence of invasive tumour; n = 3, age 5–12 years) and adults with pre-invasive TGCC (n = 4, 17–36 years) were obtained from the Erasmus MC-University Medical Centre, Rotterdam (Institutional review board—MEC 02.981 and CCR2041) and from the biobank at the Department of Growth and Reproduction, University Hospital of Copenhagen, Copenhagen, Denmark (H-1-2012-007). The presence of pre-GCNIS was determined by the expression of POU5F1. These tissues were obtained from patients with Disorders of Sexual Development (DSD), infertility, or suspected TGCC for diagnostic purposes. Details about the proteins analysed in these samples can be seen in Table A3.

Invasive TGCC tissue was obtained from clinical orchiectomy specimens from men with seminoma (n = 15) and non-seminoma (n = 14). These tissues contained regions with histologically normal spermatogenesis, GCNIS-containing tubules, or the tumour. The presence of GCNIS cells was confirmed by POU5F1 expression prior to commencing the study. Details about the proteins analysed in these samples can be seen in Table A4 and Table A5.

Additional testicular tissue from TGCC patients (n = 6) was collected to include separate biopsies from each patient. One biopsy was obtained ‘distant’ to the tumour in a region that was macroscopically normal, whilst the other biopsy was located ‘adjacent’ to the tumour. POU5F1 was used to identify tubules containing GCNIS cells within each region and co-staining for cytokeratin (‘immature’ Sertoli cells marker) and androgen receptor (‘mature’ Sertoli cell marker) was used to determine the maturity status of the Sertoli cells within these GCNIS-containing tubules. Details about the proteins analysed in these samples can be seen in Table A6 and Table A7.

### 2.2. Immunohistochemistry

Sections were dewaxed in xylene (Fisher Chemicals, Hampton, NH, USA) rehydrated in graded alcohols (Fisher Chemicals), and washed in tap water. Antigen retrieval was performed in 0.01 M citrate buffer (10 mM Citric Acid, 0.05% Tween 20, pH 6.0 with hydrochloric acid, all reagents from Sigma-Aldrich, Poole, UK) in a decloaking chamber (Biocare Medical, Berkshire, UK) as previously described [14]. Sections were then washed in tap water and endogenous peroxidase was blocked with 3% hydrogen peroxide (Merck Millipore, Bulrlington, MA, USA) in methanol (Fisher Chemicals) for 30 min, followed by two washes in Tris Buffer saline (TBS, 24 g Tris base, 77 g NaCl, pH to 7.6 with hydrochloric acid, all reagent from Sigma-Aldrich, and distilled water up to 1 L) for 5 min each. Then, avidin and biotin were blocked (Vector labs, Burlingame, CA, USA) for 15 min each, and sections were washed with TBS. Sections were blocked with normal chicken serum (NChS) for 30 min at room temperature (RT). Incubation with primary antibody was then performed overnight at 4 °C. The following day sections were washed with TBS and incubated with secondary antibody (Santa Cruz Biotechnology, Dallas, TX, USA) at 1:200 in NChS for 30 min, followed by incubation with streptavidin horse radish peroxidase (HRP; 1:1000) for 30 min. Colour visualisation was achieved after the incubation with 3’Diaminobenzidine (DAB; Vector Labs, Newark, CA, USA). Sections were then washed and dehydrated in increasing concentrations of alcohol and 100% xylene for 5 min, and coverslipped with Pertex (Histolab, Gothenburg, Sweden). Antibody details are described in Table 1. 

### 2.3. Immunofluorescence

Sections were dewaxed in xylene (Fisher Chemicals), rehydrated in graded alcohols (Fisher Chemicals), and washed in tap water. Antigen retrieval, hydrogen peroxidase blocking, serum block with NChS, and primary antibody incubation were performed as described for immunohistochemistry. The following day, sections were washed with TBS and incubated with peroxidase labelled secondary antibody (Santa Cruz Biotechnology) at 1:200 in NChS for 30 min, followed by washes in TBS and incubation with Tyramide signal amplification (TSA, Perkin Elmer, Waltham, MA, USA), at 1:50 for 10 min. Antigen retrieval was then performed by microwaving the sections at full power for 2.5 min in 0.01 M citrate buffer, followed by a 30 min cool-down period. The process from NChS block up to primary antibody detection was repeated twice more for two subsequent primary antibodies. Sections were counterstained with DAPI (4′, 6-diamidino-2-phenylindole, Sigma-Aldrich) by adding 1 µL/mL of TBS and incubating the sections for 10 min in the dark. Finally, sections were washed with TBS and mounted with PermaFluor (Life Technologies, Paisley, UK). Primary antibody details are described in Table 1. 

### 2.4. Image Capture

Immunohistochemistry was imaged with a PROVIS microscope (Olympus Optical, London, UK) and a Canon DS126131 camera (Canon, Tokyo, Japan); images were processed using the Axiovision SE64 Rel. 4.9.1 (Zeiss) software. Fluorescent images were captured on LSM710 Zeiss confocal microscope (Carl Zeiss, Hertfordshire, UK) and images were processed with the ZEN software (Carl Zeiss).

### 2.5. Quantification of Cells

For quantification of desmin expression in human fetal testis (n = 18), double immunofluorescence for SOX-9 and desmin was performed, and several images were taken depending on the size of the tissue. Details about the fetal age of included samples, the number of images taken per slide, and the number of Sertoli cells counted are included in Table 2. 

For quantification of cytokeratin expression in Sertoli cells from seminoma (n = 3) and non-seminoma (n = 3) GCNIS-containing tubules, immunohistochemistry for cytokeratin was performed and Sertoli cells were counted in tubules with disrupted spermatogenesis and morphologically visible GCNIS cells (Table 3).

For quantification of cytokeratin expression in Sertoli cells (n = 6), a double immunofluorescence for cytokeratin and androgen receptor expression on biopsies adjacent to tumour and distant to tumour was performed. Given the size of the biopsies, some tissues only contain 4 to 6 tubules, Sertoli cells were only quantified in GCNIS-containing tubules (Table 4).

Cells were counted with the Image J software (Image J, U. S. National Institutes of Health, Bethesda, MD, USA).

### 2.6. Statistical Analysis

Statistical analysis was performed using GraphPad Prism software version 6.04 (GraphPad Software Inc., La Jolla, CA, USA). Unpaired *t*-test was performed for the analysis of desmin expression in SOX-9 positive cells and desmin expression in TGCC, comparing seminoma against non-seminoma patients and for the analysis of cytokeratin^+^ Sertoli cells of GCNIS-containing tubules from seminoma and non-seminoma samples. Paired Student’s *t*-test was performed for the analysis of cytokeratin expression in biopsies ‘adjacent’ to, and ‘distant’ from the tumour.

## 3. Results

### 3.1. Expression of Cytokeratin and Desmin during Human Fetal Testis Development

**Figure 1:**
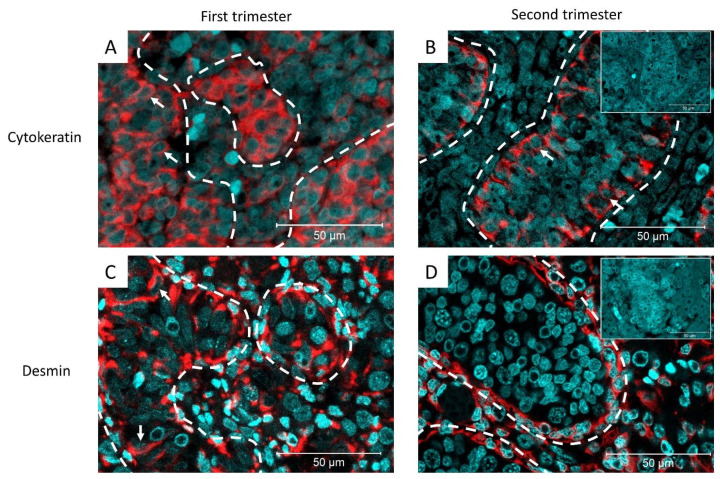
Representative images of cytokeratin expression in (**A**) first trimester (11-gestational week [GW]) and (**B**) second trimester (18-GW) of human fetal testis development. White arrows: cytokeratin+ Sertoli cells, inset: negative control (n = 8). (**C**) Desmin expression in the first trimester (10-GW) and (**D**) second trimester (17-GW) of human fetal testis development. White arrows: desmin^+^ Sertoli cells. Dashed lines represent the periphery of the seminiferous tubule. Inset: negative control (n = 22).

The expression of cytokeratin and desmin was examined at different stages of testicular development and in relation to TGCC progression. For this purpose, a pan-cytokeratin antibody, which recognizes a wide range of cytokeratins (cytokeratin 1, 4, 5, 6, 8, 10, 13, 18, and 19) was used. In human fetal testis, cytokeratin expression was observed in the Sertoli cell cytoplasm in the first and second trimesters (8–20 weeks of gestation). Desmin was also expressed in the Sertoli cell cytoplasm during the first trimester of human fetal testis development. In second-trimester tissues, desmin expression is mainly observed in interstitial cells and to a lesser extent in the Sertoli cell cytoplasm (Figure 1). 

**Figure 2:**
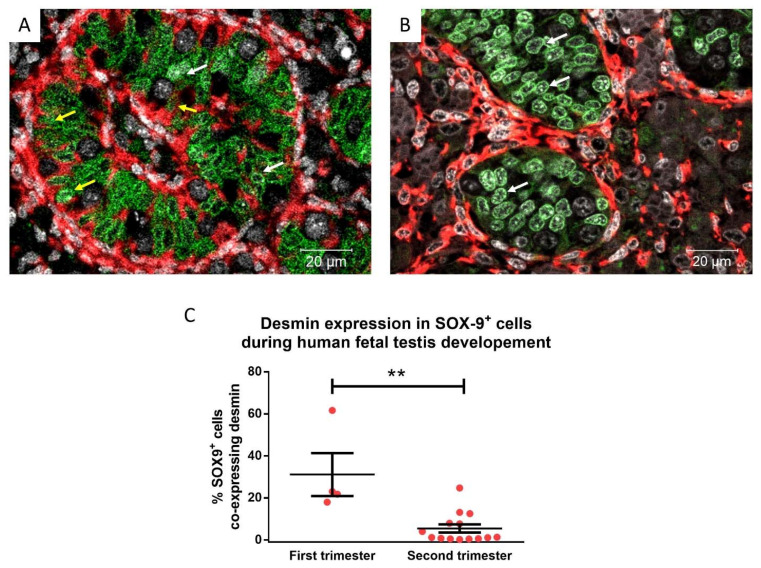
Desmin (red) and SOX-9 (green; Sertoli cells) expression in human fetal testis in first (**A**; 10 gestational weeks—GW) and second trimester (**B**; 18 GW) tissues. Yellow arrows: desmin^+^ Sertoli cells, white arrows: desmin^−^ Sertoli cells. Counterstained with DAPI (grey). **C**) Desmin expression in SOX-9^+^ cells during human fetal testis development during the first trimester (8–13 GW, n = 4) and second trimester (14–20 GW, n = 14). Mean ± SEM, analysed by unpaired *t*-test. ** *p* < 0.01.

The expression of desmin decreased with age during fetal testicular development. Therefore, the proportion of Sertoli cells (SOX-9**^+^**) co-expressing desmin in first and second-trimester tissues was quantified. There was a significant decrease (31.14% vs. 6.74%, *p =* 0.0016) in the proportion of desmin expressing Sertoli cells in the second trimester, compared to the first-trimester human fetal testis (Figure 2). 

### 3.2. Cytokeratin and Desmin Expression in Normal Adult Human Testis

**Figure 3:**
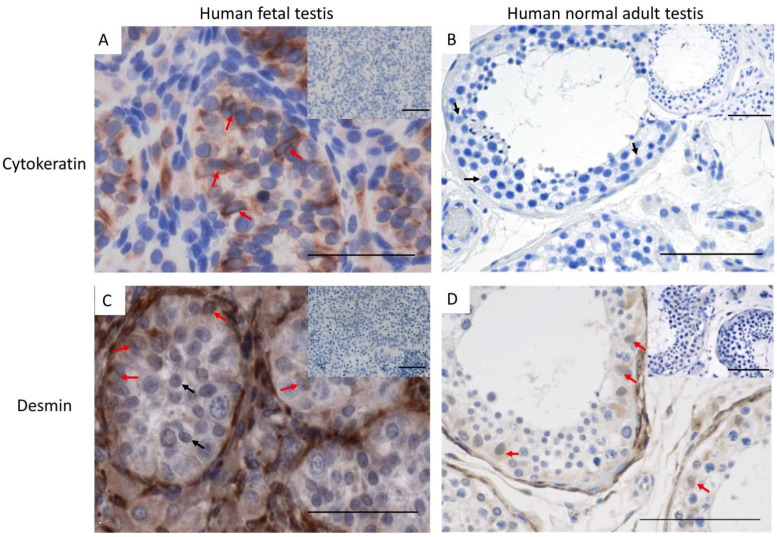
Cytokeratin expression in (**A**) human fetal testis (20 GW) and (**B**) normal adult testis (n = 3). red arrows: cytokeratin^+^ Sertoli cells, black arrows: cytokeratin^−^ Sertoli cells. (**C**) Desmin expression in normal fetal testis (17 GW) and (**D**) normal adult testis (n = 6). Scalebars represent 50 µM (**A**,**C**) and 100 µM (**B**,**D**), red arrows: desmin^+^ Sertoli cells, black arrows: desmin^−^ Sertoli cells, insets: negative controls.

Next, the expression of cytokeratin and desmin was examined in the Sertoli cells of a normal adult testis. In contrast to the human fetal testis, cytokeratin was not detected in the Sertoli cells of the normal adult testis (Figure 3A,B), suggesting that loss of cytokeratin is an indicator of Sertoli cell maturation. Desmin was detected in human fetal testis samples as well as in the normal adult testis (Figure 3C,D). 

### 3.3. Cytokeratin and Desmin Expression in Testicular Germ Cell Cancer (TGCC) Tissue

**Figure 4:**
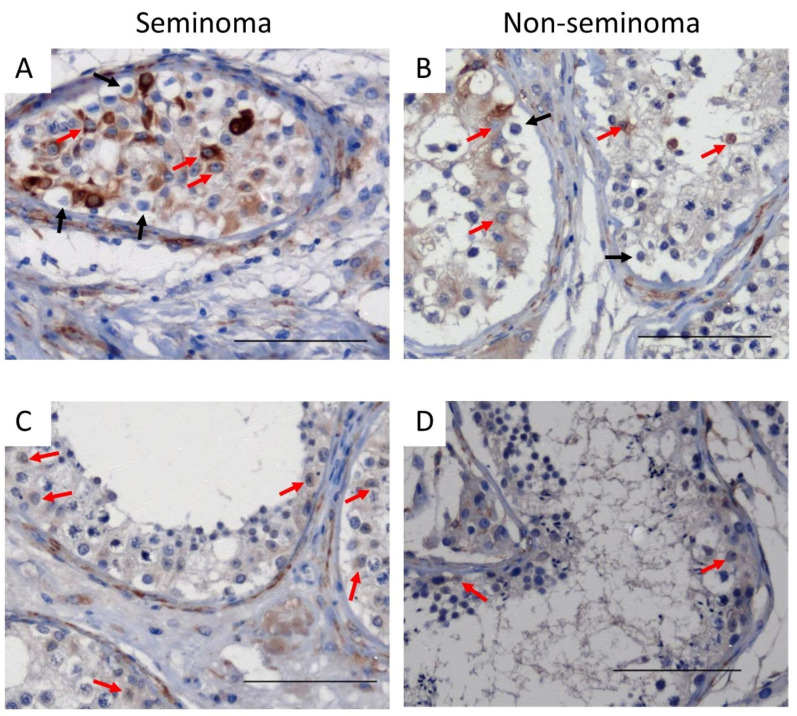
Desmin expression in Sertoli cells of (**A**,**B**) GCNIS-containing tubules and (**C**,**D**) tubules with active spermatogenesis on tissue from (**A**,**C**) seminoma and (**B**,**D**) non-seminoma patients. Scalebars represent 100 µM, red arrows: Sertoli cells, black arrows: GCNIS cells.

In TGCC samples, desmin was expressed in Sertoli cells from GCNIS-containing tubules (Figure 4A,B) and in Sertoli cells from tubules with active spermatogenesis (Figure 4C,D) from patients with either seminoma or non-seminoma (Figure 4). 

**Figure 5:**
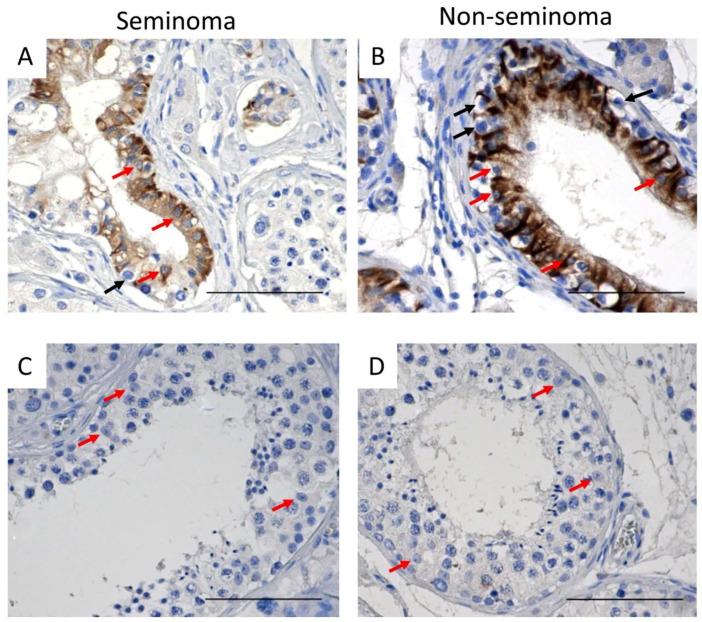
Cytokeratin expression in Sertoli cells of (**A**,**B**) GCNIS-containing tubules and (**C**,**D**) tubules with active spermatogenesis on tissue from (**A**,**C**) seminoma and (**B**,**D**) non-seminoma patients. Scalebars represent 100 µM, red arrows: Sertoli cells, black arrows: GCNIS cells.

In patients with seminoma or non-seminoma, cytokeratin was heterogeneously expressed in Sertoli cells in tubules containing GCNIS cells (Figure 5A,B) whereas, in tubules with active spermatogenesis, cytokeratin was not observed (Figure 5C,D). 

**Figure 6:**
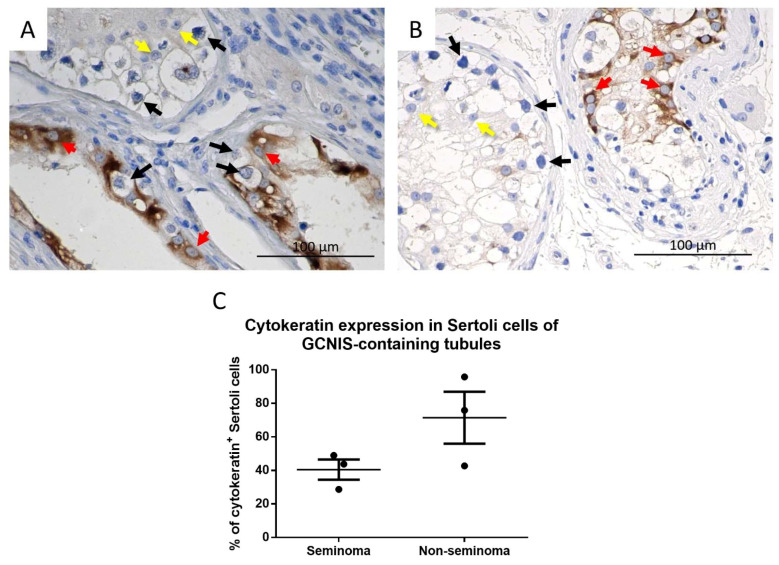
Cytokeratin expression in GCNIS containing tubules from a (**A**) seminoma and (**B**) non-seminoma patient. Red arrows: cytokeratin^+^ Sertoli cells, yellow arrows: cytokeratin^−^ Sertoli cells, black arrows: GCNIS. (**C**) Semi-quantitative analysis of cytokeratin^+^ Sertoli cells of GCNIS containing tubules from seminoma and non-seminoma patients. Mean ± SEM, data analysed by unpaired *t*-test.

Cytokeratin expression in Sertoli cells from GCNIS-containing tubules was noted to be heterogeneous with cytokeratin^+^ and cytokeratin^−^ Sertoli cells observed. In addition, some of the GCNIS-containing tubules had no cytokeratin-positive Sertoli cells (Figure 5). There was no difference in the proportion of cytokeratin^+^ and cytokeratin^−^ Sertoli between GCNIS-containing tubules from seminoma and non-seminoma samples (Figure 6C).

**Figure 7:**
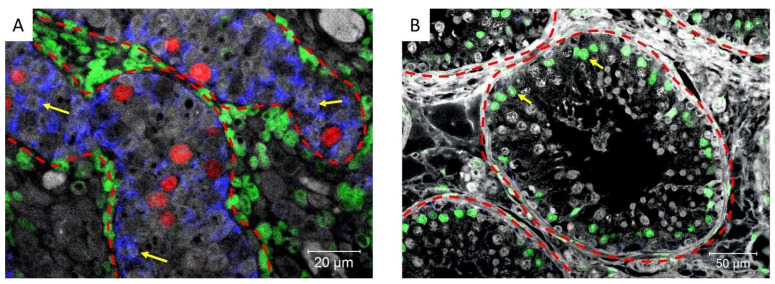
Cytokeratin (blue), Androgen receptor (green), and POU5F1 (red) expression in (**A**) an 18-week-old human fetal testis (where POU5F1 is normally expressed in fetal gonocytes) and (**B**) normal adult testis. Dashed lines represent the periphery of the tubules and yellow arrows: Sertoli cells.

We then investigated the expression of cytokeratin, a marker of immature Sertoli cells, and androgen receptor (AR) a marker of mature Sertoli cells, demonstrating that cytokeratin and AR are not co-expressed in Sertoli cells of the normal fetal or adult testis, which are Cytokeratin**^+^**/AR**^−^** (immature) and Cytokeratin**^−^**/AR**^+^** (mature), respectively (Figure 7).

**Figure 8:**
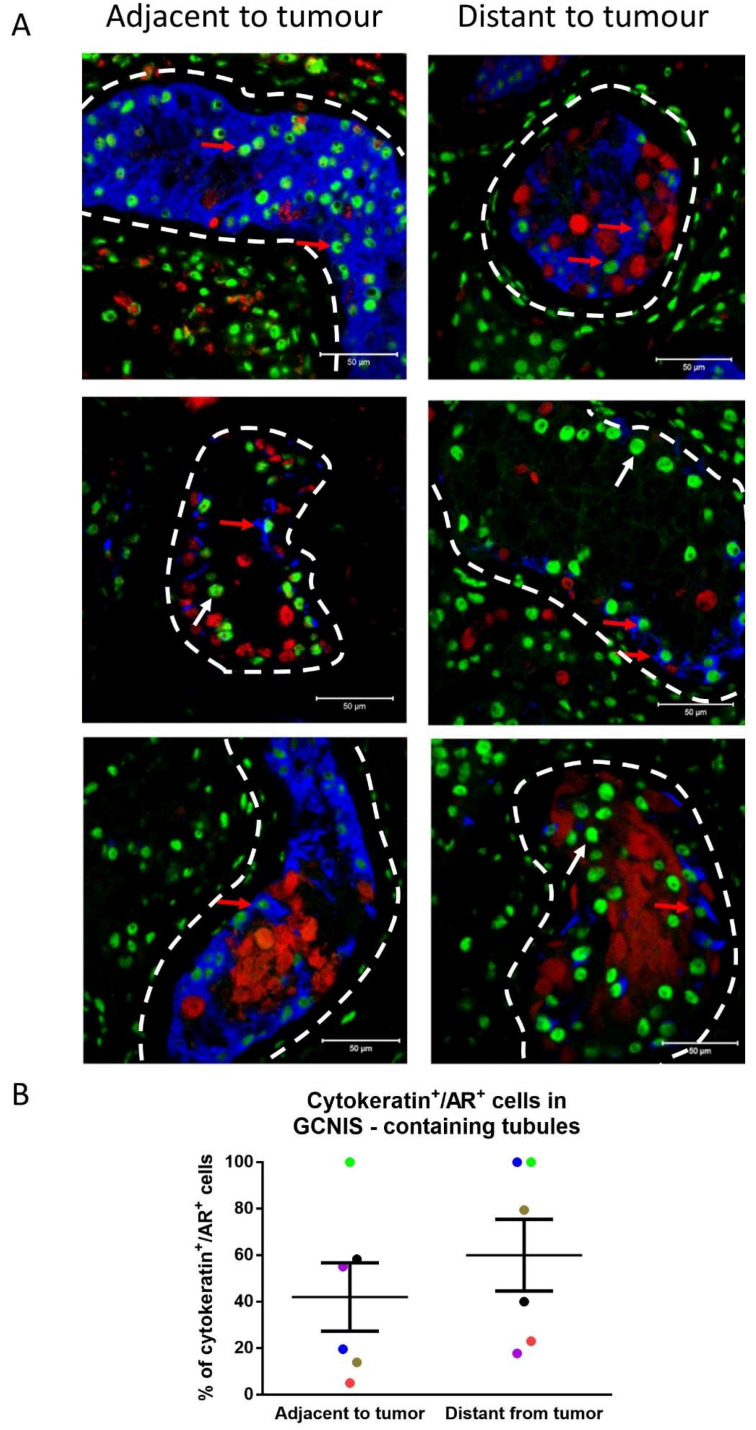
(**A**) Cytokeratin (blue), POU5F1 (GCNIS; red), and androgen receptor (AR, green) expression in Sertoli cells from GCNIS-containing tubules located distant from the tumour and adjacent to the tumour. Dashed lines represent the periphery of the seminiferous tubules. Scale bars represent 50 µm. Red arrows: cytokeratin**^+^**/AR**^+^** Sertoli cells, white arrows: cytokeratin^−^/AR**^+^** Sertoli cells. (**B**) Semiquantitative analysis of cytokeratin^+^/AR^+^ Sertoli cells in GCNIS-containing tubules adjacent and distant to the tumour (n = 6). Mean ± SEM, data analysed by paired *t*-test. Paired samples from a patient are represented by the same colour.

Based on the observed expression of cytokeratin in only a proportion of Sertoli cells in GCNIS-containing tubules within the same sample, it was evaluated whether cytokeratin expression could be associated with the location of these tubules with respect to the tumour. A co-expression of mature (AR) and immature (cytokeratin) markers in Sertoli cells of GCNIS-containing tubules was observed (Figure 8A). In GCNIS-containing tubules, from biopsies located ‘adjacent’ to the tumour and ‘distant’ to the tumour, Sertoli cells co-expressing cytokeratin and AR were quantified. There was no difference in the proportion of these cells located ‘adjacent’ to the tumour, compared with GCNIS-containing tubules ‘distant’ to the tumour (Figure 8B). 

Cytokeratin expression in testicular tissues from patients with pre-invasive disease. 

**Figure 9:**
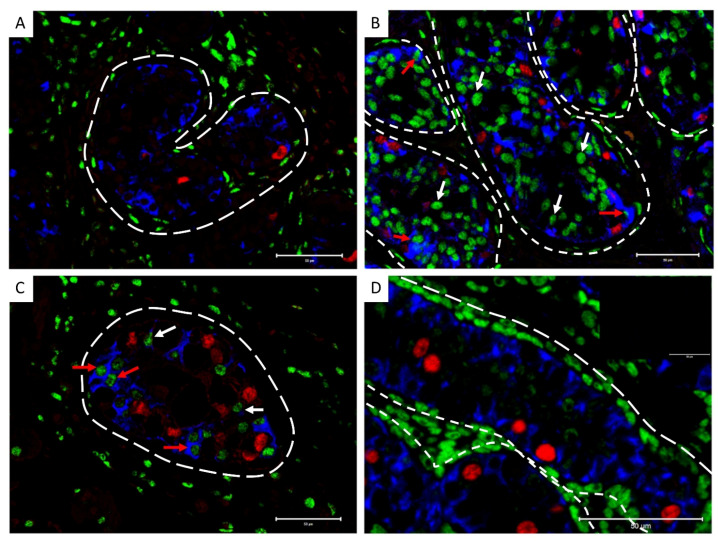
Cytokeratin (Blue), POU5F1 (GCNIS; red), and androgen receptor (AR, green) expression in GCNIS-containing tubules from childhood (**A**—7 years old, **B**—12 years old) and adult (**C**—28 years old) patients with pre-invasive TGCC. (**D**) Human fetal testis (18 GW) as a positive control for markers with negative control as inset, dashed lines represent the periphery of the seminiferous tubules. Red arrows: cytokeratin^+^/AR^+^ Sertoli cells, white arrows: cytokeratin^−^/AR^+^ Sertoli cells. Scale bars represent 50 µm.

Based on the observed cytokeratin expression in GCNIS-containing tubules from patients with invasive disease, we investigated whether cytokeratin expression could also be observed in Sertoli cells in tubules containing GCNIS cells from children and adults (n = 7; 7–36 years) with pre-invasive TGCC. Cytokeratin expression was observed in Sertoli cells of GCNIS-containing tubules, including a population of Sertoli cells that co-expressed androgen receptor and cytokeratin in both childhood and adult patients whereas in human fetal testis tissues and prepubertal samples, androgen receptor expression was not detected in Sertoli cells (Figure 9).

## 4. Discussion

The present study describes the expression of the intermediate filaments, desmin, and cytokeratin, in human Sertoli cells during normal testicular development and in human Sertoli cells from TGCC tissue from patients with pre-invasive and invasive disease. Desmin expression was observed in a varying proportion of Sertoli cells from all samples examined regardless of the stage of normal testicular development or progression to TGCC. 

The observed decrease in desmin expression during fetal development has not been reported before, however, its expression has been observed in some Sertoli cells up to 14 weeks of development [9].

It has been previously shown that desmin expression can be detected in GCNIS-containing tubules from TGCC patients [9], however, in the present study we demonstrate that desmin is also expressed in tubules from healthy adult men and tubules with active spermatogenesis from patients with invasive TGCC, therefore desmin expression does not appear to be associated with Sertoli cell maturation nor progression towards TGCC.

It has been shown that cytokeratin expression can be observed during fetal testis development up to gestational week 20 [15]. In the present study, cytokeratin expression was found in Sertoli cells of the human fetal testis at all ages studied (9–20 weeks of development), whilst in normal adult testes, cytokeratin expression was not observed. Previous reports demonstrate that cytokeratin 8 and 18 are not detected in normal adult testis [9,12,16]. In the present study, we use a pan-cytokeratin antibody that recognizes a wide range of cytokeratins (cytokeratin 1, 4, 5, 6, 8, 10, 13, 18, and 19) and we confirm the absence of cytokeratin in normal adult testis Sertoli cells.

In TGCC, cytokeratin expression was observed in GCNIS-containing tubules from patients with pre-invasive and invasive TGCC. Similar expression profiles have been reported but only in tubules near the tumour [9]. Here we report cytokeratin expression in GCNIS-containing tubules from patients with pre-invasive and invasive TGCC, where the expression in Sertoli cells in GCNIS-containing tubules is not dependent on the location of the tubules with respect to the tumour. 

The finding that cytokeratin is only present in Sertoli cells from GCNIS-containing tubules in adults with TGCC, suggests a potential use for cytokeratin as an indicator of impaired somatic cell development. 

Cytokeratin persistence might be indicative of an early feature associated with somatic cell dysfunction and its expression has also been observed in patients with cryptorchid testis and men with spermatogenic arrest, azoospermia, atrophic seminiferous tubules, and TGCC [9,11,12,16]. These features are also described as part of a Testicular Dysgenesis Syndrome (TDS), which is believed to result from impaired androgen production or action during a critical period of fetal testis development, known as the Masculinization Programming Window [17]. Early somatic cell dysfunction is also described in patients with genetic Disorders of Sex Development (DSD) in which XY individuals with gonadal dysgenesis have reduced production of Sertoli cells hormones, such as AMH and inhibin B, as well as an increased risk of TGCC, reviewed in [18,19]. 

In the present study, we demonstrate that Sertoli cells are immature in both pre-invasive and invasive TGCC samples, however, more studies are needed to determine the timing of cytokeratin expression during normal testicular development in postnatal and childhood periods in order to determine if cytokeratin persistence could be a sensitive marker of pre-invasive TGCC.

It has previously been established that cytokeratin expression is found in Sertoli cells from 12 weeks to 20 weeks of gestation [9] and that androgen receptor expression in Sertoli cells begins at 4 months of age (2–15% of Sertoli cells), progressively increasing thereafter [20,21,22]. Therefore, co-expression of cytokeratin and androgen receptors is not expected at any stage during normal testis development. However, the present study found co-expression of cytokeratin and androgen receptors in Sertoli cells of GCNIS-containing tubules from patients with pre-invasive and invasive TGCC. Thus, suggesting that either (1) Sertoli cells are only partially differentiated in TGCC patients and retain some immature features or (2) the loss of normal germ cells could have led to partial de-differentiation of Sertoli cells. In a study involving the co-culture of adult Sertoli cells with seminoma tumour cells, Sertoli cells begin to express cytokeratin after 2 weeks of co-culture [23], suggesting that the interaction between Sertoli cells and a tumour microenvironment can lead to dedifferentiation of Sertoli cells.

Future studies could also be performed to investigate the association between additional Sertoli cell proteins of intermediate filaments with pre-invasive and invasive TGCC. This would include AMH (Sertoli cells hormone) and vimentin (intermediate filament), both of which have also been associated with Sertoli cell maturation status [9,15,24,25]. In addition, the expression of extracellular matrix components (e.g., fibronectin) involved in the polarization of Sertoli cells, which is required for blood testis barrier formation, could also be explored in tissues with pre-invasive GCNIS and invasive TGCC [26].

## 5. Conclusions

Although desmin does not appear to be a reliable indicator of Sertoli cell maturation or progression towards TGCC, cytokeratin may be a useful indicator of impaired Sertoli cell maturation and progression to pre-invasive and invasive TGCC.

## Figures and Tables

**Table 1:** Summary of Primary antibodies for immunohistochemistry and immunofluorescence.

Antibody	Species Raised	Dilution for IHC	Dilution for IF	Clonality	Cat. No.	Manufacturer
Androgen receptor	rabbit	N/A	1:3000	Poly-clonal	ab74272	Abcam Ltd. (Cambridge, UK)
Pan-cytokeratin (cytokeratin 1, 4, 5, 6, 8, 10, 13, 18, and 19)	mouse	1:3000	1:5000	Mono-clonal	C2562	Sigma-Aldrich (Poole, UK)
Desmin	rabbit	1:3500	1:3500	Poly-clonal	ab15200	Abcam Ltd. (Cambridge, UK)
POU5F1	goat	N/A	1:150	Poly-clonal	sc-8626	Santa Cruz (Heidelberg, Germany)
SOX-9	rabbit	N/A	1:5000	Poly-clonal	A5535	Merk Millipore (Hertfordshire, UK)

IHC: Immunohistochemistry, IF: Immunofluorescence, N/A: not applicable as it was not used for that technique.

**Table 2:** Details of human fetal testis tissue used for semi-quantification of desmin expression in SOX-9 positive cells.

Sample Number	Age	Number of Images	Total Counted Sertoli Cells	
HFT1	8 GW	2	655	First trimester
HFT2	10 GW	3	1212	
HFT3	11 GW	2	718	
HFT4	12 GW	1	233	
HFT11	15 GW	2	772	Second trimester
HFT12	15 GW	1	456	
HFT15	16 GW	3	1318	
HFT16	16 GW	2	467	
HFT21	17 GW	2	628	
HFT22	17 GW	1	404	
HFT23	17 GW	1	335	
HFT27	18 GW	2	711	
HFT28	18 GW	2	998	
HFT29	18 GW	1	454	
HFT30	18 GW	3	1478	
HFT32	19 GW	2	602	
HFT33	19 GW	1	384	
HFT35	20 GW	4	1619	

GW: Gestational week.

**Table 3:** Details of the tissue used for the quantification of cytokeratin expression in Sertoli cells of GCNIS containing tubules from seminoma (SEM) and non-seminoma (NS) patients.

Sample Number	Number of Images	Total Counted Sertoli Cells	TGCC Type
SEM4	3	182	seminoma
SEM9	2	95	
SEM15	2	124	
NS1	2	98	non-seminoma
NS9	3	244	
NS6	2	182	

**Table 4:** Details of the tissue used for the quantification of cytokeratin expression in Sertoli cells of GCNIS containing tubules from biopsies collected adjacent to, and distant from the tumour.

	Adjacent to Tumour			Distant to Tumour	
Sample ID	Number of Images	Total Counted Sertoli Cells	Sample ID	Number of Images	Total Counted Sertoli Cells
BA4	1	20	BD4	2	270
BA5	3	299	BD5	1	22
BA6	2	142	BD6	2	90
BA8	3	169	BD8	2	132
BA11	2	63	BD11	3	46
BA14	2	55	BD14	2	144

## Data Availability

The data support the findings of this study are available upon reasonable request from the corresponding author, but restrictions apply to the availability to these data, which were used under license for the current study, and so are not publicly available.

## Appendix A

**Table A1:** Immunohistochemistry and Immunofluorescence performed on human fetal testis samples.

Human Fetal Testis Tissue Samples						
		ICH or IF Performed				
Sample ID	GW	Desmin/ SOX9	Desmin/ POU5F1	Desmin/ SOX9	Cytokeratin/ AR/POU5F1	Cytokeratin
HFT1	8	X				
HFT2	10	X				
HFT3	11	X			X	
HFT4	12	X				
HFT5	12+6	X	X			X
HFT6	13			X		X
HFT7	14		X			
HFT8	14		X			
HFT9	14		X			
HFT10	15	X				
HFT11	15	X				
HFT12	15	X				
HFT13	15				X	
HFT14	15+2		X			
HFT15	16	X				
HFT16	16	X				
HFT17	16	X				
HFT18	16		X			
HFT19	16		X			
HFT20	16+4	X				
HFT21	17	X				
HFT22	17	X				
HFT23	17	X				
HFT24	17		X			
HFT25	17		X			
HFT26	17					X
HFT27	18	X				
HFT28	18	X				
HFT29	18	X				
HFT30	18	X				
HFT31	18				X	
HFT32	19	X				
HFT33	19	X				
HFT34	19				X	
HFT35	20	X				
HFT36	20					X

IHC: Immunohistochemistry, IF: Immunofluorescence, AR: Androgen receptor

**Table A2:** Immunohistochemistry and Immunofluorescence performed on normal adult testis samples.

Normal Adult Testis Samples				
	IHC or IF Performed			
Sample ID	Desmin	Desmin/ SOX9	Cytokeratin/ AR/POU5F1	Cytokeratin
NAT1			X	
NAT2			X	
NAT3	X	X		X
NAT4	X			
NAT5	X			

IHC: Immunohistochemistry, IF: Immunofluorescence, AR: Androgen receptor.

**Table A3:** Immunohistochemistry and Immunofluorescence performed on childhood and adult pre-invasive TGCC samples.

Childhood and Adult Pre-Invasive TGCC Samples				
		IHC of IF Performed		
Sample ID	Age	Desmin	Cytokeratin/ AR/POU5F1	Cytokeratin
PR1	5		X	
PR2	7		X	
PR3	12	X	X	
PR4	17			X
PR5	26			X
PR6	28		X	
PR7	36	X		

IHC: Immunohistochemistry, IF: Immunofluorescence, AR: Androgen receptor.

**Table A4:** Immunohistochemistry and Immunofluorescence performed on non-seminoma samples.

Seminoma Samples					
	IHC or IF Performed				
Sample ID	Desmin/ SOX9	Desmin/ POU5F1	Desmin	Cytokeratin/ AR/POU5F1	Cytokeratin
S1		X	X		
S2			X		
S3		X			
S4					X
S5		X			
S6			X		
S7			X		
S8		X	X		
S9	X		X		X
S10	X	X			
S11		X			
S12		X		X	
S13		X			
S14			X		
S15					X

IHC: Immunohistochemistry, IF: Immunofluorescence, AR: Androgen receptor

**Table A5:** Immunohistochemistry and Immunofluorescence performed on non-seminoma samples.

Non-Seminoma Samples					
	IHC or IF Performed				
Sample ID	Desmin/ SOX9	Desmin/ POU5F1	Desmin	Cytokeratin/ AR/POU5F1	Cytokeratin
NS1					X
NS2			X		
NS3			X		
NS4	X		X		
NS5			X	X	X
NS6					
NS7			X		
NS8		X			
NS9		X	X		X
NS10		X	X		X
NS11		X	X		
NS12			X		
NS13			X		
NS14	X				

IHC: Immunohistochemistry, IF: Immunofluorescence, AR: Androgen receptor

**Table A6:** Immunohistochemistry and Immunofluorescence performed on biopsies obtained in areas ‘distant’ to the seminoma tumour.

Biopsies ‘Distant’ from the Tumour		
	IHC or IF Performed	
Sample ID	Desmin	Cytokeratin/AR/POU5F1
BD1	X	
BD2	X	
BD3	X	
BD4		X
BD5		X
BD6		X
BD7		X
BD8		X
BD9		X
BD10		X
BD11		X
BD12		X
BD13		X
BD14		X

IHC: Immunohistochemistry, IF: Immunofluorescence, AR: Androgen receptor

**Table A7:** Immunohistochemistry and Immunofluorescence performed on biopsies obtained in areas ‘adjacent’ to the seminoma tumour.

Biopsies ‘Adjacent’ from the Tumour	
	IHC or IF Performed
	Cytokeratin/AR/POU5F1
BA15	X
BA5	X
BA6	X
BA7	X
BA8	X
BA11	X
BA14	X
BA4	X

IHC: Immunohistochemistry, IF: Immunofluorescence, AR: Androgen receptor
